# Compounds Involved in the Invasive Characteristics of *Lantana camara*

**DOI:** 10.3390/molecules30020411

**Published:** 2025-01-19

**Authors:** Hisashi Kato-Noguchi, Midori Kato

**Affiliations:** Department of Applied Biological Science, Faculty of Agriculture, Kagawa University, Miki 761-0795, Kagawa, Japan

**Keywords:** allelochemical, β-caryophyllene, herbivore, invasive species, lantadene, natural enemy, nematode, pathogen

## Abstract

*Lantana camara* L. is native to tropical America and has naturalized in many other tropical, subtropical, and temperate regions in Asia, Africa, Oceania, North and South America, and Europe. *L. camara* infests diverse habitats with a wide range of climatic factors, and its population increases aggressively as one of the world’s 100 worst invasive alien species. Its infestation reduces species diversity and abundance in the natural ecosystems and reduces agricultural production. The life history characteristics of *L. camara*, such as its high reproductive ability and high adaptive ability to various environmental conditions, may contribute to its ability to infest and increase its population. Possible evidence of the compounds involved in the defense functions of *L. camara* against natural enemies, such as herbivore mammals and insects, parasitic nematodes, pathogenic fungi and bacteria, and the allelochemicals involved in its allelopathy against neighboring competitive plant species, have accumulated in the literature over three decades. Lantadenes A and B, oleanonic acid, and icterogenin are highly toxic to herbivore mammals, and β-humulene, isoledene, α-copaene thymol, and hexadecanoic acid have high insecticidal activity. β-Caryophyllene and *cis*-3-hexen-1-ol may function as herbivore-induced plant volatiles which are involved in sending warning signals to undamaged tissues and the next plants of the same species. Farnesol and farnesal may interrupt insect juvenile hormone biosynthesis and cause abnormal metamorphosis of insects. Several triterpenes, such as lantanolic acid, lantoic acid, pomolic acid, camarin, lantacin, camarinin, ursolic acid, and oleanonic acid, have demonstrated nematocidal activity. Lantadene A, β-caryophyllene, germacrene-D, β-curcumene, eicosapentaenoic acid, and loliolide may possess antimicrobial activity. Allelochemicals, such as caffeic acid, ferulic acid, salicylic acid, α-resorcylic acid, *p*-hydroxybenzoic acid, vanillic acid, unbelliferone, and quercetin, including lantadenes A and B and β-caryophyllene, suppress the germination and growth of neighboring plant species. These compounds may be involved in the defense functions and allelopathy and may contribute to *L. camara*’s ability to infest and to expand its population as an invasive plant species in new habitats. This is the first review to focus on how compounds enhance the invasive characteristics of *L. camara*.

## 1. Introduction

**Figure 1:**
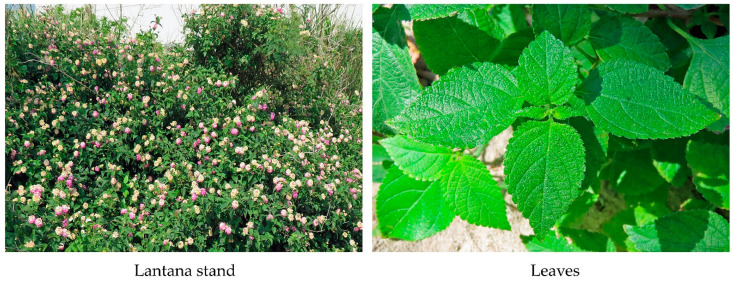
Stand and leaves of *L. camara*.

*Lantana camara* L., belonging to the Verbenaceae family, is a perennial shrub, 1–4 m in height, with quadrangular or cylindrical stems and small prickles. Multiple stems frequently arise at the base of the main stems and form dense impenetrable stands. The oval leaves are opposite and serrate [1,2,3,4] (Figure 1). The native range of the species is tropical America, and it was introduced into many counties as an ornamental plant [5,6,7]. *L. camara* has spread across tropical, subtropical, and temperate regions in Asia, Africa, Oceania, North and South America, and Europe [1,2,3,8].

*L. camara* infestations were monitored in 134 plots from 1997 to 2008 in the Biligiri Rangaswamy Temple Wildlife sanctuary (540 km^2^) in the Western Ghats of India. *L. camara* occupied 41% and 81% of the plots in 1997 and 2008, respectively, and the mean density of *L. camara* stands increased 4-fold from 1997 to 2008 [9]. The total area of infestation of *L. camara* in India has already covered 13 million ha [10]. In South Africa, the species occupied 25,000–30,000 ha in 1962 and 2.2 million ha in the early 1980s [7,11]. The species occupied more than 5 million ha of coastal and sub-coastal Queensland and NSW in Australia. Its distribution has been expanding from the Torres Strait throughout eastern Queensland to the southern border of NSW and Western Australia, the Northern Territory, and Victoria [12]. Considering the risk of its invasiveness, *L. camara* has been listed in the world’s 100 worst invasive alien species by IUCN [13].

A global spatial invasion risk assessment of *L. camara* using a random forest modeling approach showed that *L. camara* has already infested and/or established itself in 114 countries. The areas with a high invasion risk were estimated to be between 35° N and 35° S latitude, and an additional 27 countries are at risk of serious invasion [14]. The global climate change warming trend increases the risk of invasion of *L. camara* in all continents except Antarctica [14,15,16]. The risk of invasion of Europe will increase by 251%, compared to current levels, from 2061 to 2080, and 28 countries will face a risk increase of over 50% [14].

*L. camara* causes significant impacts on natural ecosystems and agricultural production [5,11]. Infestation of *L. camara* interrupts the regeneration process of native plant species and lowers species richness and density in native plant communities, including in endangered plant species. It was observed that juvenile plant densities of native plant species were lower in areas infested with *L. camara* than in non-infested areas [9,17,18,19,20]. *L. camara* was reported to occupy 23% of the total area of protected areas (1633 ha of 7006 ha) of the Lehri Nature Park in Pakistan [21], and it has already infested many other protected areas [9,22,23,24]. The species has adversely affected 1300 native species, including endangered plant species in Australia and Hawaii [2,25].

The infestation of *L. camara* also affects soil properties, increasing total carbon and phosphate concentrations and soil moisture [26,27]. The modification of the soil properties affects species composition and ecosystem structure and functions, resulting in alteration of food webs and the habitats of vertebrate and invertebrates [28]. *L. camara* infestation lowers the abundance and richness of invertebrates, birds, and mammals [29,30,31,32,33]. In addition, *L. camara* is easy to burn, and increases fire risk in dry rainforests by shifting the distribution of available fuel beds and providing a more continuous fuel layer [34].

*L. camara* infestation causes a major problem in most agricultural areas. Its large, impenetrable thickets reduce grass abundance and species in pastures and block the movement of domestic stocks to waterholes [8]. *L. camara* has the negative effects on the farm operation and production. The infestation caused the reduction in maize and cassava (main crops in the area) yield by 26–50%, forage availability by over 50%, and medicinal plant availability in eastern Africa [35]. The species causes significant reductions in coconut plantations in the Philippines, Fiji, Solomon Islands, and Vanuatu [36,37], oil palm and rubber plantations in Malaysia, banana plantations in Australia and Samoa [8], citrus plantations in Florida [38], tea plantations in India and Indonesia [39], and timber plantations in Australia, South Africa, India, and Indonesia [39,40,41]. The considerable resources are spent on *L. camara* management. The control annual cost was estimated to be ca. INR 9000 per hectare (reported in 2009) in India [42], R 1.7 million (reported in 1999) in South Africa, and AUD 104 million (reported in 2007) in Australia only for stock-farming industry [7,8].

*L. camara* shows high genetic variations and high levels of adaptive ability to various environmental conditions [6,43,44]. *L. camara* infests into diverse habitats with a wide range of climatic factors. *L. camara* generally grows well in open habitats, such as sparse forests, forest margins, grasslands, riparian zones, and coastal plains. It also infests into the disturbed areas, such as forestry plantations, orchards, agriculture fields, abandoned land, roadsides, and railroads [5,11,19,20]. *L. camara* grows in tropical, subtropical, and temperate climates, with mean annual precipitation ranging from 4000 mm to 1000 mm, at 13 °C for the lower limit of the mean temperature. However, the species tolerates the annual precipitation as low as 200 mm and at 0 °C for the absolute minimum temperature [2,5]. *L. camara* can grow on a variety of soil types, pH values between 4.5 and 8.5, nutrient-rich to nutrient-poor soil, moist to dry soil, or sandy soil to clay soil [6,28].

**Figure 2:**
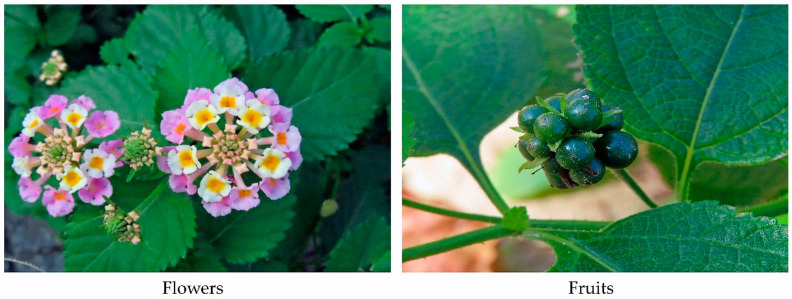
Flowers and fruits of *L. camara*.

*L. camara* grows and flowers throughout the year under good growing conditions [5,6,10] and produces 12,000 fruits, containing seeds, per plant in each year [28,45]. The seeds are mainly dispersed by birds and water flow, including flooding water [5,6]. The seed viability was reported to be ranging from 2 to 5 years, and the germination rate was 20–50% under both laboratory and field conditions [6,28]. However, the germination rate increased after the birds feeding because of the digestion of seed pulp [6,8]. *L. camara* also reproduces asexually. The regeneration occurs through the ramet sprouting. The ramets arise from the dormant buds at the bases of the stems [28]. These observations suggest that *L. camara* has the high reproductive adaptivity and high adaptive ability to various environmental conditions [6,28]. Such characteristics of the life history may contribute to the infestation and expanding population of *L. camara* in the introduced ranges (Figure 2).

Several review articles have summarized the biology, impacts, and management of *L. camara* [5,6,7,8,9,10,11,28,30,46]. Other review articles have summarized the biologically active compounds of *L. camara* for the purpose of the development of medicines and pesticides [47,48,49,50,51,52,53,54]. However, there has been no review article focusing on the compounds involved in the invasive characteristics of *L. camara*. This is the first review article providing an overview of the compounds involved in the invasive characteristics of *L. camara* and the action mechanisms of these compounds. The literature was searched using a combination of the predominant online search engines: Scopus, ScienceDirect, and Google Scholar and all possible combinations of *L. camara* with the following words: invasiveness, adaptively, ecology, habitat, reproduction, impact, natural enemy, herbivore, nematode, insecticidal activity, fungicidal activity, symbiosis, pharmacology, allelopathy, allelochemical, and secondary metabolite.

## 2. Defense Compounds Against Herbivore Mammals

The ingestion of *L. camara* plants causes critical symptoms of photosensitivity, hepatomegaly, jaundice, and nephrosis in herbivore mammals, including livestock such as cattle, sheep, goats, rabbits, buffaloes and horses [48,55]. The incidents of *L. camara* poisoning occur when adequate fodder supply is limited during drought and flooding conditions and when animals are transported from *Lantana*-free areas to *Lantana*-infested areas [56,57,58]. After feeding on the leaves, stems, and/or roots of *L. camara*, these animals suffer from constipation in 2 h, stasis in 4–6 h, photosensitization in 24–48 h, and then swelling of their muzzles and eyelids. Toxic substances, such as lantadene A, lantadene B, lantadene C, lantadene D, oleanonic acid, and icterogenin in *L. camara* plants, are absorbed through the gastrointestinal tracts of these animals and transported to the liver in portal blood. Then, these toxic substances cause cholestasis and hepatotoxicity, and induce hepatic necrosis [59]. These compounds also cause jaundice and nephrosis. It was observed that 74 of 170 heifers were dead in a few weeks after the feeding of *L. camara* [59], and 40% of mice were dead [60]. The severity of the symptoms depends on the quantity of foliage taken [57,58]. The LD_50_ value of the partially purified lantadene powder gave to sheep by intravenous injection was 1–3 mg/kg body weight, while the LD_50_ value of the lantadene powder by oral administration was 60 mg/kg body weight [61]. Lantadene A and lantadene B are the major constituents among these toxic substances in *L. camara*, and lantadene A is responsible for most of the toxic effects [58,62]. Lantadene A and lantadene B are metabolized into toxic derivatives in vivo, named as reduced lantadene A and reduced lantadene B, respectively [62].

**Figure 3:**
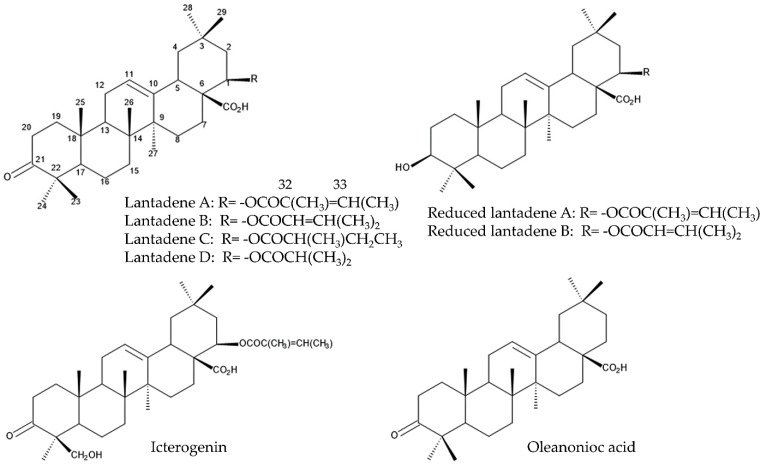
The compounds involved in the defense function against herbivore mammals.

Chemical structures of lantadenes and reduced lantadenes have been determined and are shown in Figure 3 [63,64,65,66]. Lantadenes A, B, C, and D are similar structures, except for the side chain at C1 position. Atoms C32 and C33 on the side chain are connected by a double bond in lantadenes A and B and by a single bond in lantadenes C and D. The different structures of the side chain of lantadenes A, B, C, and D may affect the toxicity [63,64,66].

*Lantana* toxicity was reported to be caused through the alteration of the microsome and mitochondrial structures in the liver cells. *Lantana* leaf powder decreased the protein contents, phospholipid-to-protein ratio, and cholesterol-to-protein ratio in the hepatic microsomes, while the ratio of cholesterol to phospholipid did not change [48,67], suggesting the dissociation of endoplasmic reticulum fragments from the microsomes. *Lantana* leaf powder also decreased protein contents in the hepatic mitochondrial protein. The activities of glutamate dehydrogenase, succinic dehydrogenase, Mg^2+^-ATPase, and cytochrome oxidase of the mitochondria were increased, while the activity of NADH–ferricyanide reductase remained unchanged [48]. Lantadene A and reduced lantadene A also affect the hepatic mitochondrial membranes, and reduced lantadene A acts as a mitochondrial uncoupler of oxidative phosphorylation, resulting in decreasing ATP levels in the liver cells [68]. Caspase 3 was detected in the hepatocytes in lantadene-poisoned animals via immunostaining [59]. Caspase 3 is an important regulator and indicator of the apoptosis [69,70]. Thus, lantadene toxicity may cause the apoptosis in the hepatocytes, and the apoptosis plays a significant role in the pathogenesis of *Lantana* poisoning.

*L. camara* infestation decreased the species abundance and diversity of herbivore mammals in the Groenkloof Nature Reserve in South Africa. The species abundance and diversity of carnivore mammals also decreased as those of the herbivores decreased [32]. The avoidance of *L. camara* stands by rodents, such as *Lemniscomys rosalia*, *Mastomys coucha*, and *Saccostomus campestris*, was observed in the Groenkloof Nature Reserve [32]. These observations suggest that *L. camara* produces the toxic substances, protects form the feeding of herbivorous mammals, and affects the species abundance and diversity of the herbivorous and carnivorous mammals.

## 3. Defense Compounds Against Herbivorous Insects

The feeding activity of herbivorous insects often causes serious damages to the growth, development, and regeneration processes of several plant species [71,72,73]. Therefore, many plant species have developed protective traits against herbivorous insect attacks [74,75,76,77]. It was also reported that many invasive plant species produce certain compounds, which have the defense functions against herbivorous insects [75,78,79].

During the field surveys for the natural enemies of *L. camara* in its native ranges, the leaf-feeding mirid, *Falconia intermedia*, was identified as a potential biocontrol agent of *L. camara*. *Falconia intermedia* is one of the most abundant and damaging natural enemies of *L. camara* in the native ranges [80,81]. Therefore, *Falconia intermedia* was released as a biocontrol agent of *L. camara* in South Africa in 1999 and Australia in 2000 [82,83]. Larvae and adults of *Falconia intermedia* feed on the leaves of *L. camara* and cause severe chlorosis, defoliation, and a reduction in the flowering. The lifetime of the adults is about three weeks, and the females lay 2–3 eggs per day on the undersides of the leaves of *L. camara*. The development of the larvae is completed in 20–25 days [83,84]. *Falconia intermedia* initially colonized well in the *L. camara* stands, and rapidly increased its population size. However, the population of *Falconia intermedia* disappeared within a few years at almost all sites in South Africa [82]. The attempt of the biological control of *L. camara* using *Falconia intermedia* also resulted in very little success in Australia [8].

*L. camara* induces the physical and chemical defense responses within eight weeks after the insect damage caused by *Falconia intermedia* [85,86]. *L. camara* increases its leaf toughness and trichome density on the newly developed leaves [85]. Leaf toughness depends on the deposition of lignin, cellulose, suberin, sclerencyma fibers, xylem, and collenxhymas, and the tough leaves can protect from the attacks of the insect feedings. The trichomes prevented attacks from the insect feeding and oviposition [87,88,89,90].

*L. camara* also increased 2.5-fold in the emission of β-caryophyllene after the feeding of *Falconia intermedia* [86]. β-Caryophyllene has been reported to act as the herbivore-induced plant volatiles (HIPVs) in other several plant species, and the emission of β-caryophyllene increased in response to the attacks of herbivorous insects [91,92,93,94,95,96]. *cis*-3-Hexen-1-ol is one of the major constituents in the essential oil of *L. camara* [94], and is also known to act as HIPV [95]. The different bouquets of HIPVs (chemical competition and concentration) are emitted by several plant species following the herbivore feeding [95,96].

The emission of HIPVs works for the plants in two ways [95,96,97]. (1) The chemical signals from the damaged plant tissues to the undamaged tissues of the damaged plants and to the adjacent same plant species: Undamaged tissues of the plants and neighboring plants can realize tomorrow’s herbivorous insect attacks and prepare the physical defense responses, such as the increasing leaf toughness and trichome density. (2) HIPVs attract and stimulate the predator insects to hunt the herbivorous insects as their prey. The predator insects receive HIPVs by the olfactory sensilla in their antennae [96]. The action of the predator insects to HIPVs is different from the predator species, and only particular HIPVs can stimulate the hunting behavior of the given predators. The stimulated hunting behavior of the predators reduces number of the herbivorous insects, and further feeding damages [95,96,97]. Therefore, the increasing emission of β-caryophyllene with *cis*-3-hexen-1-ol may be involved in the indirect defense responses, avoiding the feeding damages by the herbivorous insects.

The ethanol extracts of *L. camara* leaves increased the mortality of the moth larvae of the cotton leafworm *Spodoptera litura* [98] and another moth larvae of the cabbage cluster caterpillar *Crocidolomia pavonana* [99]. The LC_50_ values of the extracts for *Spodoptera litura* were 16,347 ppm and 3548 ppm at 24 h and 48 h after the extract application, respectively [98]. The *n*-hexane extracts of *L. camara* leaves increased the mortality of the larvae of the red cotton stainer *Dysdercus koenigii* and also cause its abnormal metamorphosis from the larvae to adult insects. The active compounds were identified as β-caryophyllene and hexadecanoic acid through a GC-MS analysis [100]. Hexadecanoic acid has been reported for its antibacterial activity [101]. Farnesol and farnesal were also identified in the extracts of *L. camara* leaves [100]. Farnesol and farnesal are the intermediates of the insect juvenile hormone biosynthesis pathway via mevalonic acid. The juvenile hormone is involved in the insect metamorphosis [102,103]. Application of the extracts may disturb the juvenile hormone biosynthesis and cause the abnormal metamorphosis of the larvae [100]. In addition, the *n*-hexane extracts of *L. camara* leaves interrupt the mating behavior of the adults of the red cotton strainer and suppress the egg hatching [104].

**Figure 4:**
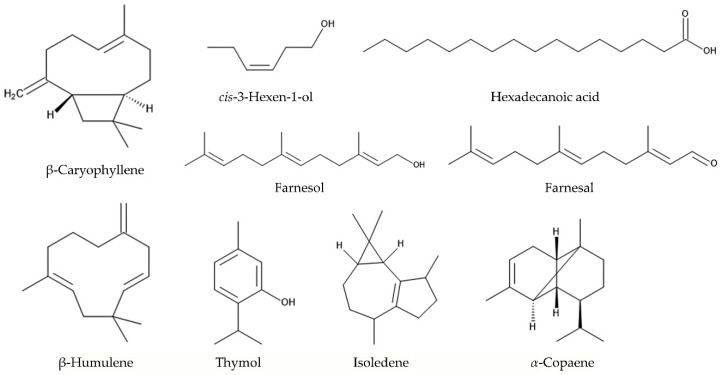
The compounds involved in the defense function against herbivorous insects.

The essential oil of *L. camara* showed the insecticidal and insect repellent activity against the adults of the grain weevil *Sitophilus granariesm*, and the major constituents in the essential oil were β-caryophyllene, β-humulene, and thymol [105]. The *n*-hexane extracts and the essential oil of *L. camara* showed the insecticidal activity against the storage grain pests, such as the bean weevil *Callosobruchus maculatus*, the maize weevil *Sitophilus zeamais* [106,107], the wheat weevil *Sitophilus granarius* [108], the beetle *Tribolium castaneum*, the cigarette beetle *Lasioderma serricorne*, and the adzuki bean weevil *Callosobruchus chinensis* [109,110]. β-Caryophyllene (70%), isoledene (12%), and α-copaene (4.1%) were the major constituents in the essential oil [110]. Incorporation of chipped fresh leaves and stems of *L. camara* into soil prevented the attacks of the *Coptotermes formosanus* and *Reticulitermes flavipes* termites [111]. In addition, *L. camara* infestation decreased the species abundance and diversity of invertebrates, including insects, in the Groenkloof Nature Reserve in South Africa [24] (Figure 4).

One of the essential factors for the success of the invasive plant species infestation in the introduced ranges is its defense ability against the herbivorous insects [71,72,73,74,75]. Specific monophagous herbivores for certain invasive plant species may be few in the introduced ranges because there is no co-evolutional history between the invasive plants and herbivorous insects in the introduced ranges [112]. It is not clear if all of the insects described in this Section feed on *L. camara* in the introduced ranges. However, the compounds, which have the insecticidal activity, may contribute to the protection from the feeding activity of the herbivorous insects and contribute to the invasive characteristics of *L. camara*.

## 4. Defense Compounds Against Parasitic Nematodes

The parasitic nematodes make galls in the host plant roots and deprive photosynthate and other nutrients from their host plants. This parasitism causes a significant growth restraint in the host plant species and the reduction in the plant vigor and defense ability against other pathogen attacks [113,114,115,116]. Root-knot nematodes *Meloidogyne* spp. are distributed worldwide, and the host range of their parasitism is wide. Therefore, *Meloidogyne* spp. are considered to be one of the major pathogenic nematodes [117,118].

The aqueous extracts of *L. camara* leaves increased the mortality of *Meloidogyne incognita* and significantly suppressed its egg hatching and root-knot development [119,120]. The aqueous extracts and decomposing leaves of *L. camara* also suppressed the population density of *Meloidogyne javanica* and its root-knot development [121,122]. The aqueous extracts of *L. camara* roots mixed into soil resulted in increasing the mortality of *Meloidogyne javanica* in the soil and in decreasing its egg hatching [123,124]. These observations suggest that *L. camara* contains certain compounds, which have nematicidal activity.

**Figure 5:**
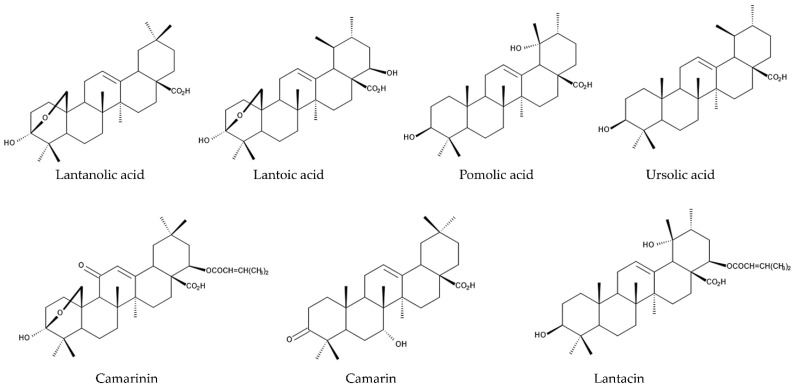
The compounds involved in the defense function against parasitic nematodes.

Seven triterpenes, such as lantanolic acid, lantoic acid, pomolic acid, ursolic acid, camarinin, camarin, and lantacin, were isolated from aerial parts of *L. camara* as nematocidal active substances. Lantanolic acid, lantoic acid, and pomolic acid showed 100% mortality of *Meloidogyne incognita* at a 1 mg/mL concentration after 24 h application, while ursolic acid, camarinin, camarin, and lantacin exhibited 100% mortality of *Meloidogyne incognita* at 1 mg/mL concentration after 48 h application [125,126]. Oleanonic acid was also isolated from aerial parts of *L. camara* and showed nematocidal activity [127,128]. Therefore, these compounds may be involved in the nematicidal activity of the extracts of *L. camara*. In addition, *L. camara* infestation decreased species abundance and diversity of nematodes in the Groenkloof Nature Reserve in South Africa [24] (Figure 5).

## 5. Defense Compounds Against Pathogenic Fungi and Bacteria

The plant pathogenic fungi are divided into two groups: (1) biotrophic pathogens, which persist in plants and deprive nutrient from plants, and (2) necrotrophic pathogens, which kill the tissue to extract nutrients from plants. Necrotrophic pathogens cause necrosis and even death of the infected plants [129,130,131]. *Fusarium* is widely distributed in soil, and some of *Fusarium* spp. cause the *Fusarium* diseases, such as wilt, blight, rot, canker, and root necrosis on the host plant species in both agricultural and natural ecosystems. *Fusarium oxysporum* and *Fusarium solani* are known to be necrotrophic fungus species [132]. The essential oil of *L. camara* suppressed the growth of *Fusarium oxysporum* and *Fusarium solani* [133,134]. The main constituents of the essential oil were germacrene-D (19.55%), β-caryophyllene (17.53%), and β-curcumene (10.22%) [134]. The aqueous and aqueous ethanol extracts of *L. camara* leaves suppressed the growth of *Fusarium oxysporum* [135,136]. Aqueous methanol extracts of *L. camara* leaves also suppressed the *Fusarium oxysporum* and *Fusarium solani*. Lantadene A was identified as the active principal of the extracts [137].

The essential oil of *L. camara* suppressed the growth of other pathogenic fungi, *Corynespora cassiicola, Rhizoctonia solani, Agroathelia rolfsii*, and *Alternaria brassicicola* [133,138]. The main constituents of the essential oil were germacrene-D (19.8%) and β-caryophyllene (19.7%) [138]. *Corynespora cassiicola* has a wide range of host plant species and causes foliar spots, defoliation, and debilitation to the infected plants [139,140,141]. *Rhizoctonia solani* is facultative with a wide host range and worldwide distribution. It causes various plant diseases, such as root rot, damping off, and wire stems [142]. *Agroathelia rolfsii* (syn. *Sclerotium rolfsii*) usually occurs in soil as a saprotroph but can attack living plants. It has an indiscriminate host range and causes blight and root rot [143]. *Alternaria brassicicola* is a fungal necrotrophic plant pathogen and causes black spot disease on a wide range of plant species [144].

**Figure 6:**
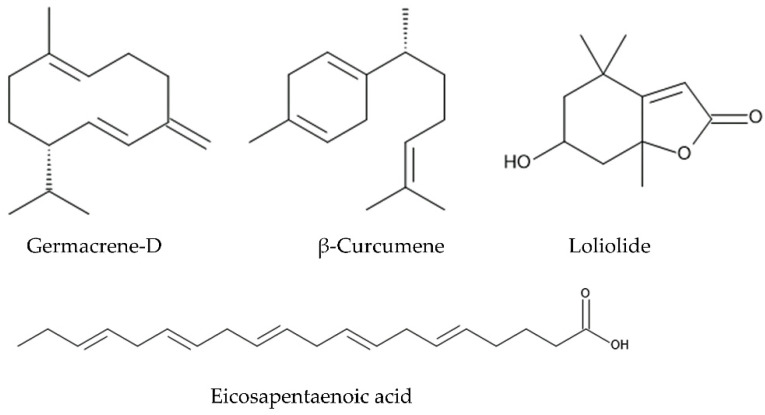
The compounds involved in the defense function against pathogenic fungi and bacteria.

The methanol extracts of *L. camara* leaves and flowers suppressed the growth of the pathogenic bacterium *Xanthomonas axonopodis*. The inhibitory activity of extracts between the leaves and flowers were not clearly different [145]. *Xanthomonas axonopodis*, belonging to the gamma subdivision of Proteobacteria, infects diverse plant hosts and causes canker and necrotic lesions [146]. Through a molecular docking analysis, eicosapentaenoic acid and loliolide were suggested to be active ingredients of the extracts [145]. It was also reported that *L. camara* alters the soil microbial communities in the infested areas. The altered soil microbial community, including beneficial symbiosis microbes, enhances the growth and stress tolerance of *L. camara* [147,148]. Some other invasive plant species, such as *Imperata cylindrica*, *Mimosa pigra*, *Chromolaena odorata*, *Fallopia japonica*, and *Ageratum conyzoides*, were reported to alter the soil microbial communities, including rhizobium and AMF communities, by releasing certain compounds and to create a better soil microbial community for these invasive plant species but not for the native plant species. The created soil microbial communities enhance the growth and stress tolerance of these invasive plants [149,150,151,152] (Figure 6).

The observations suggest that *L. camara* produces the compounds, such as lantadene A, germacrene-D, β-caryophyllene, β-curcumene, eicosapentaenoic acid, and loliolide. These compounds protect it from the infection of the pathogenic fungi and bacteria and may contribute to the invasive characteristic of *L. camara*.

## 6. Compounds Involved in Allelopathy

Many invasive plants show relatively high allelopathic activity and release allelochemicals into the neighboring environment, including their rhizosphere soil [153,154,155]. Allelochemicals interrupt the germination, growth, and development of the neighboring plant species. Consequently, these invasive plant species acquire stronger competitive ability against the neighboring plant species and obtain a relatively large quantity of resource, such as nutrients, water, and light in their local plant communities [156,157,158]. The ability of the invasive plant species in the resource acquisition is one of the important factors for their infestation success in the introduced ranges [159,160,161,162,163]. Allelochemicals are synthesized, stored in the certain plant parts, and released through volatilization, root exudation, and decomposition of plant parts in the rhizosphere soil [164,165]. Therefore, allelochemicals have been identified in the extracts of the certain plant tissues (leaves, stems, and roots), volatiles, essential oil, root exudation, and rhizosphere soil [166,167,168].

Aqueous leaf extracts of *L. camara* inhibited the germination of *Cucumis sativus*, *Phaseolus mungo*, *Raphanus sativus*, *Vigna unguiculate*, *Cicer arietinum* [169], and *Capsicum annuum* and *Daucus carota* [170]. The aqueous leaf extracts also caused the necrosis of the leaves of *Eichhornia crassipes* [171] and interrupted the regeneration process of the moss *Funaria hygrometrica* [172]. Methanol extracts of the stem and leaves of *L. camara* suppressed the germination and growth of *Lolium multiflorum* [173]. Soaking water of the *L. camara* leaves, stem, and/or flowers suppressed the germination and growth of *Mimosa pudica* [174,175]; *Pennisetum americanum*, *Setaria italica*, and *Lactuca sativa* [176]; *Triticum aestivum* [177,178]; and *Cucurbita pepo*, *Phaseolus vulgaris*, and *Lycopersicon esculentum* [179] and killed *Eichhornia crassipes* [180,181]. These observations suggest that the extracts of *L. camara* possess the allelopathic activity, and *L. camara* contains certain extractable allelochemicals. Some of these allelochemicals may be released from *L. camara* plants into the neighboring environments by rain and irrigation water.

When the seeds of *Triticum aestivum*, *Zea mays*, *Glycine max*, *Lepidium virginicum*, and *Abutilon theophrasti* were sown into the mixture of the chopped shoots of *L. camara* and sand, the growth of these seedlings was significantly suppressed [182]. The decomposed residues of the roots, leaves, and/or shoots of *L. camara* also suppressed the growth of *Morrenia odorata* [183], *Urena lobate, Bidens bipinnata*, and *Bidens pilosa* [184]. Rhizosphere soil of *L. camara* inhibited the growth of *Achyranthes aspera* and *Albizia lebbeck* [185] and the germination and growth of *Avena sativa*, *Cicer arietinum*, *Hordeum vulgare*, and *Triticum aestivum* [186]. These observations suggest that certain allelochemical may be released during the decomposition process of *L. camara* plants, and the rhizosphere soil may contain the allelochemicals. These allelochemicals may also be released into the rhizosphere soil through the root exudation and/or the leachate from the living plants by rain and irrigation water.

**Figure 7:**
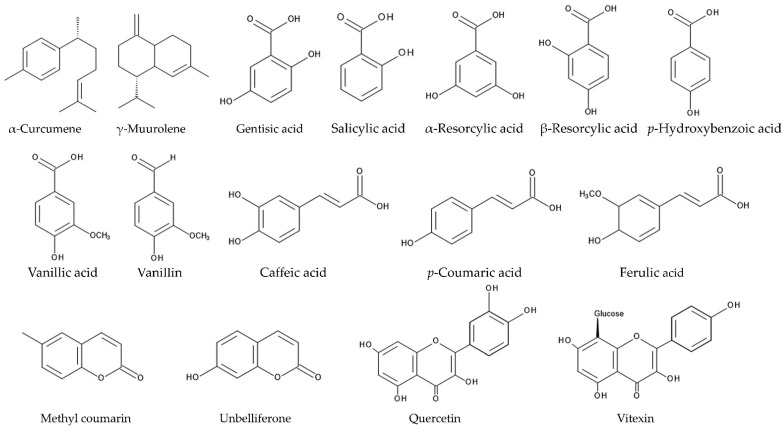
The compounds involved in the allelopathy.

Lantadene A and lantadene B were found in the rhizosphere soil of *L. camara* as allelochemicals. Lantadene A and lantadene B inhibited the growth of *Eichhornia crassipes* and *Microcystis aeruginosa* at concentrations greater than 13.7 mg/L and 10.8 mg/L, respectively [187]. The essential oil obtained from *L. camara* leaves also suppressed the seedling growth of *Portulaca oleracea*. The major compounds of the essential oil were α-curcumene, β-caryophyllene, and γ-muurolene [188] (Figure 7).

Caffeic acid, *p*-coumaric acid, ferulic acid, gentisic acid, salicylic acid, α-resorcylic acid, β-resorcylic acid, *p*-hydroxybenzoic acid, vanillic acid, vanillin, methyl coumarin, unbelliferone, and quercetin were identified in the aqueous leaf extracts of *L. camara* as allelochemicals [189]. Although all the compounds inhibited the root and shoot growth of *Lolium multiflorum*, the inhibitory activity of unbelliferone, methyl coumarin, and salicylic acid was higher than other compounds [189]. A flavonoid, quercetin, was also identified in some other plant species as an allelochemical and suppressed the growth of several pant species and the mitochondrial function [190,191,192]. A flavone glucoside, vitexin, was isolated from the *L. camara* leaves as an allelochemical [193] (Figure 7).

Benzoic acid and cinnamic acid derivatives, such as caffeic acid, *p*-coumaric acid, ferulic acid, gentisic acid, salicylic acid, α-resorcylic acid, β-resorcylic acid, *p*-hydroxybenzoic acid, vanillic acid, and vanillin, were identified in a wide range of plant extracts and plant rhizosphere soil [156,194,195,196]. The involvement of these compounds in plant allelopathy and their mode of actions have been investigated in many other plant species [196,197,198]. These compounds are synthesized from phenylalanine in the shikimic acid pathway and affect the constitution of lipids and proteins of the plasma membranes of the plant cells [199,200]. Consequently, the plasma membranes lose the transmembrane electrochemical potential, and the depolarization of the membranes occurs. The depolarization causes a nonspecific efflux of both anions and cations, including magnesium, nitrate, potassium, and phosphate ions, and changes water balance in the cells. These compounds were also reported to disturb various enzyme activities involved in the phytohormone synthesis, photosynthesis, protein synthesis, and the secondary metabolites and to interrupt the plant cell division and plant growth and development [196,197,198,199,200]. Therefore, those compounds found in *L. camara* may affect the structure of the plasma membranes, the transmembrane electrochemical potential, and several enzyme activities involved in the essential metabolism in the plant cells.

Aqueous leaf extracts of *L. camara* increased reactive oxygen species (ROS) and decreased catalase activity in *Eichhornia crassipes* leaves, causing the occurrence of the necrosis of the leaves [171]. Catalase converts ROS to molecular oxygen and hydrogen peroxide and reduces the oxygen stress conditions [201,202,203]. Thus, the extracts of *L. camara* cause oxidative stress conditions and interrupt the enzyme activity for the ROS extinction. Anther invasive plant species, *Mikania micrantha*, was also reported to increase the ROS in the neighboring plant species due to the emission of β-caryophyllene. β-Caryophyllene causes the oxidative stress conditions [204]. β-Caryophyllene was also identified in the essential oil of *L. camara* [105,134,138,188]. These observations suggest that β-caryophyllene in the extracts of *L. camara* may cause oxidative stress conditions, and interrupt ROS extinction, resulting in the necrosis of the neighboring competitive plant species.

These observations suggest that *L. camara* is allelopathic and produces and releases certain allelochemicals into the neighboring environments, including its rhizosphere soil. These released allelochemicals suppress the germination, growth, and/or development of the competitive plant species. According to the novel weapon hypothesis, the allelochemicals released from the invasive plant species are more effective in the introduced ranges than in the native ranges of the invasive plant species. The plant species in the native ranges of the invasive plant species may have obtained the ability to cope with the inhibitory effects of these allelochemicals during their co-evolutional history. However, the competitive plant species in their introduced ranges may not have an opportunity to obtain the tolerance because they have not been existing together [159,160]. Therefore, allelochemicals of *L. camara* may be effective in the introduced ranges and contribute the infestation and expanding the population of *L. camara*.

## 7. Contributions of the Compounds to the Invasive Characteristics of *L. camara*

*L. camara* produces several defensive compounds against the herbivore mammals and insects, parasitic nematodes, pathogenic fungi and bacteria, and allelochemicals against the neighboring competitive plant species. Among them, lantadene A and lantadene B showed hepatotoxic activity against herbivore mammals through the interruption of the hepatic microsome and mitochondrial functions [48,59,67,68]. Lantadene A also showed anti-fungal activity against the pathogenic fungi *Fusarium* spp. and allelopathic activity against *Eichhornia crassipes* and *Microcystis aeruginosa* [137,187].

*L. camara* emits β-caryophyllene and *cis*-3-hexen-1-ol upon herbivorous insect attacks, and these compounds may work as the HIPVs involved in the indirect defense functions: (1) chemical signals from damaged plant tissues to undamaged tissues and (2) attractions of the predator insects to hunt the herbivorous insects [95,96,97]. β-Caryophyllene also showed insecticidal activity and fungicidal activity [100,105,134]. Farnesol and farnesal may disturb the insect juvenile hormone biosynthesis and cause the abnormal metamorphosis of the insects [100,101,102,103,104].

**Figure 8:**
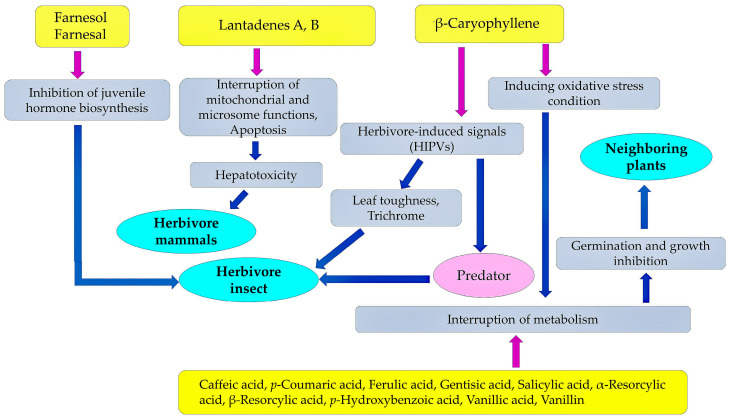
Action mechanisms of the compounds discussed in this paper. These compounds are involved in the hepathoxic, insecticidal, nematocidal, fungicidal, and allelopathic activity of *L. camara*. Purple arrow: direct action; blue arrow: secondary and tertiary action.

Lantanolic acid, lantoic acid, pomolic acid, camarin, lantacin, camarinin, ursolic acid, and oleanonic acid showed nematocidal activity, increasing the mortality and suppressing the egg hatching of the nematodes [125,126,127,128]. Germacrene-D and β-curcumene suppressed the growth of pathogenic fungi [134,138]. β-Caryophyllene, α-curcumene, β-curcumene, γ-muurolene, caffeic acid, *p*-coumaric acid, ferulic acid, gentisic acid, salicylic acid, α-resorcylic acid, β-resorcylic acid, *p*-hydroxybenzoic acid, vanillic acid, vanillin, methyl coumarin, unbelliferone, quercetin, and vitexin may act as allelochemicals, causing the inhibition of the germination and growth of neighboring competitive plant species [188,189,193]. These compounds are summarized in Table 1, and the action mechanisms of some of the compounds for the defense functions and allelopathy are shown in Figure 8.

Phytochemical and pharmacological investigations showed that *L. camara* contains many other secondary metabolites in several chemical classes, such as monoterpenes, sesquiterpenes, triterpenes, and flavonoids. Some of these compounds have been reported to exhibit pharmacological activities, such as anticancer activity, anti-inflammatory activity, anti-viral activity, antipyretic activity, and wound-healing activity for medicinal treatments, anti-microbial activity for food security, and anti-mosquito activity [47,48,49,50,51,52,53,54]. Although these biological active compounds have not yet been connected to the invasive characteristics of *L. camara*, some of them may be involved in the invasive characteristics of *L. camara* regarding its unknown functions. Several authors have suggested that *L. camara* contains alkaloids [145,205,206]. They detected alkaloid in the extracts of *L. camara* using Harbore method or Mayer’s reagent, which make the precipitation of alkaloid [207,208]. Many alkaloids are toxic and act as defensive compounds for several plant species against herbivores and pathogenic fungi [209,210,211]. However, no specific alkaloid compound has been reported in *L. camara*.

## 8. Conclusions

*L. camara* has naturalized in many tropical, subtropical, and temperate regions as one of the world’s 100 worst invasive alien species. Its infestation reduces the species diversity and abundance in the natural ecosystems, as well as agricultural production. The life history characteristics of *L. camara*, such as its high reproductive ability and high adaptive ability to various environmental conditions, may contribute to its ability to infest and increase its population. In addition, *L. camara* produces several compounds involved in defense functions against their natural enemies, such as herbivore mammals and insects, parasitic nematodes, and pathogenic fungi and bacteria, as well as allelochemicals involved in the allelopathy against competitive plant species. Their defense functions against natural enemies, as well as allelopathy against the competitive plant species, are among the essential factors that are necessary for success in dealing with the infestation and expanding population. Therefore, these compounds may also contribute to the infestation and expansion of *L. camara* in the new habitats.

## Figures and Tables

**Table 1:** Compounds involved in the defense functions against natural enemies, such as herbivore mammals and insects, parasitic nematodes, and pathogenic fungi and bacteria, as well as allelochemicals involved in the allelopathy.

		Defense Function Against				Allelopathy	Reference
Phytochemical Class	Compound	Mammal	Insect	Nematode	Fungus, Bacterium	Competitive Plant	
Triterpene	Lamtidines A	✓			✓	✓	e.g., [59,62,68,134,187]
	Reduced lantadene A	✓					[62,68]
	Lantadene B	✓				✓	[58,62,187]
	Reduced lantadene B	✓					[62]
	Lantadene C	✓					[59]
	Lantadene D	✓					[59]
	Icterogenin	✓					[59]
	Oleanonic acid	✓		✓			[59,127,128]
	Lantanolic acid			✓			[125,126]
	Lantoic acid			✓			[125,126]
	Pomolic acid			✓			[125,126]
	Ursolic acid			✓			[125,126]
	Camarinin			✓			[125,126]
	Camarin			✓			[125,126]
	Lantacin			✓			[125,126]
Sesquiterpene	β-Caryophyllene		✓		✓	✓	[86,100,105,134,188]
	β-Humulene		✓				[105]
	Isoledene		✓				[110]
	α-Copaene		✓				[110]
	Farnesol		✓				[100]
	Farnesal		✓				[100]
	Germacrene-D				✓		[134,138]
	β-Curcumene				✓		[134]
	α-Curcumene					✓	[188]
	γ-Muurolene					✓	[188]
Monoterpene	Thymol		✓				[105]
	Loliolide				✓		[145]
Aromatic compound	Methyl coumarin					✓	[189]
	Unbelliferone					✓	[189]
Phenolic acid	Salicylic acid					✓	[189]
	Gentisic acid					✓	[189]
	*p*-Hydroxybenzoic acid					✓	[189]
	Vanillic acid					✓	[189]
	α-Resorcylic acid					✓	[189]
	β-Resorcylic acid					✓	[189]
Phenolic aldehyde	Vanillin					✓	[189]
Phenylpropanoid	Caffeic acid					✓	[189]
	*p*-Coumaric acid					✓	[189]
	Ferulic acid					✓	[189]
Flavonoid	Quercetin					✓	[189]
Flavone glucoside	Vitexin					✓	[193]
Alcohol	*cis*-3-Hexen-1-ol		✓				[94]
Fatty acid	Hexadecanoic acid		✓				[100]
	Eicosapentaenoic acid				✓		[145]

## Data Availability

Not applicable.
